# Peritoneal Tuberculosis Mimicking Ovarian Malignancy: A Case Report

**DOI:** 10.7759/cureus.76231

**Published:** 2024-12-22

**Authors:** Eman A Al-zughali, Nashaat A Al-Shami, Anas W Hamedat, Salwa Al-Bustanji, Motasem Almaletti, Eishah M Al-shaibani, Saleh A Ba-shammakh

**Affiliations:** 1 Department of Internal Medicine, The Islamic Hospital, Amman, JOR; 2 Department of Gastroenterology and Hepatology, The Islamic Hospital, Amman, JOR; 3 Department of General Surgery, The Islamic Hospital, Amman, JOR; 4 Department of Radiology, The Islamic Hospital, Amman, JOR; 5 Department of General Surgery, Ministry of Health, Amman, JOR

**Keywords:** abdominal tuberculosis, caseating granulomas, ovarian masses, peritoneal tuberculosis (tb), primary extrapulmonary tuberculosis

## Abstract

Peritoneal tuberculosis (TB) is a rare extrapulmonary form of TB that often mimics ovarian malignancy, posing diagnostic challenges. This report presents a 16-year-old Jordanian female with abdominal distension, weakness, anorexia, and night sweats. Initial imaging, including contrast-enhanced computed tomography (CT), revealed compartmentalized ascites, peritoneal thickening, and enlarged ovaries with masses, suggesting possible ovarian malignancy. Laboratory findings showed elevated C-reactive protein (CRP) and cancer antigen 125 (CA-125) levels. The ascitic fluid analysis demonstrated high protein content with a neutrophil predominance, while pelvic magnetic resonance imaging (MRI) suggested tuberculous peritonitis. Exploratory laparotomy identified intra-abdominal adhesions, omental nodules, and a thickened peritoneum. Histopathology confirmed TB through granulomatous inflammation with caseating granulomas. This case underscores the importance of including TB in the differential diagnosis of ovarian malignancy, especially in TB-endemic areas, and highlights the role of early diagnosis and multidisciplinary management.

## Introduction

Tuberculosis (TB) stands as a significant global public health challenge caused by *Mycobacterium tuberculosis* (*M. tuberculosis*), which has the potential to infect various tissues and organs within the human body. This health concern is particularly pervasive in poor nations, carrying with it substantial stigma and misconceptions. Recognized as the “second great imitator,” TB has the remarkable capability to mimic other medical conditions, often causing delays in both seeking care and reaching a timely diagnosis [1]. Risk factors for developing active TB include immunocompromising conditions, such as human immunodeficiency virus (HIV), undernutrition, diabetes mellitus, smoking, heavy alcohol use, end-stage renal disease (ESRD), and certain medications [2].

Within the spectrum of extrapulmonary manifestations, peritoneal TB accounts for a modest 2% of cases [3]. People with peritoneal TB often exhibit symptoms that closely resemble those seen in individuals with cancer or who have recently undergone abdominal surgery. This is because the inflammatory changes associated with peritoneal TB can be non-specific and overlap with other medical conditions. Distinguishing between different causes becomes crucial during the initial stages of diagnosis to avoid delays in treatment and unnecessary tests or procedures. The preferred method for confirming the diagnosis is a laparoscopic biopsy, which is considered the gold standard in this context [4].

In over 90% of cases where patients are diagnosed with peritoneal TB, they initially present with ascites. The remaining 10% exhibit a more advanced stage with a “dry and doughy” abdomen. Additionally, abdominal pain and fever are prevalent symptoms found in most of these cases. These symptoms collectively play a crucial role in recognizing and understanding peritoneal TB during its early stages. The critical factor in enhancing patient outcomes for this disease lies in achieving a timely diagnosis. However, in female patients, the diagnostic process becomes particularly challenging, especially when the initial symptoms strongly suggest a gynecological malignancy [5].

This emphasizes the need for a thorough and broad approach to understanding the condition better. It is important because things can get complicated, and symptoms may overlap. Taking this approach helps to pinpoint the exact issue more accurately and quickly.

## Case presentation

A 16-year-old Jordanian female presented with a chief complaint of abdominal distention lasting for one week. The patient reported experiencing generalized weakness, a noticeable decrease in appetite, and episodes of excessive sweating that primarily occurred at night. There was no significant past medical or surgical history, nor any notable family history of TB or allergies.

On clinical examination, the patient was conscious, alert, and fully oriented. Her vital signs were recorded: a body temperature of 37.4 °C, heart rate of 118 beats per minute (bpm), respiratory rate of 18 breaths per minute, blood pressure of 118/82 mmHg, and an oxygen saturation level of 97% at room air. The patient weighed 50 kilograms, with a body mass index (BMI) of 22 kg/m². The abdomen was distended up to the xiphisternum, with no palpable masses or organomegaly. Additionally, there was no evidence of lymphadenopathy or any other systemic involvement.

The biochemical evaluation revealed a reduced hemoglobin level of 10.28 g/dL and a normal total leukocyte count of 6,300/mm³. C-reactive protein (CRP) was significantly elevated at 155.5 mg/L (Table 1).

**Table 1:** Laboratory results HB: hemoglobin, WBC: white blood cells, CRP: C-reactive protein, RBS: random blood sugar, LFT: liver function test, KFT: kidney function test, PT: prothrombin time, INR: international normalized ratio, aPTT: activated partial thromboplastin time

Laboratory test	Result	Reference range
WBC count	6.3	(4-11) x 10^3^/µL
Neutrophils	70%	40-75%
Lymphocytes	20%	20-45%
Monocytes	4%	2-10%
Eosinophils	0.2%	1-6%
Basophils	*	0-1%
HB	10.2	M:(13-17) g/dL
PCV	32	M:(40-50)%
RBC COUNT	4.7	M:(4.5-5.5) x 10^6^/µL
MCV	69	80-96 fL
MCH	22	27-32 pg
MCHC	33	32-35%
RDW	19	11.6-14%
Platelets count	652	(150-450) * 10^3^/µL
RBS	100	RR UP to 199 mg/dL
CRP	155	Less than 5
ESR	40	Less than 50y: up to 15 mm/h
Pt	14.2	13.2 sec
INR	1	-
PTT	31.9	26-36 sec
Calcium	8.8	8.6-10.2 mg/dL
LFT	Normal	-
KFT	Normal	-

**Figure 1:**
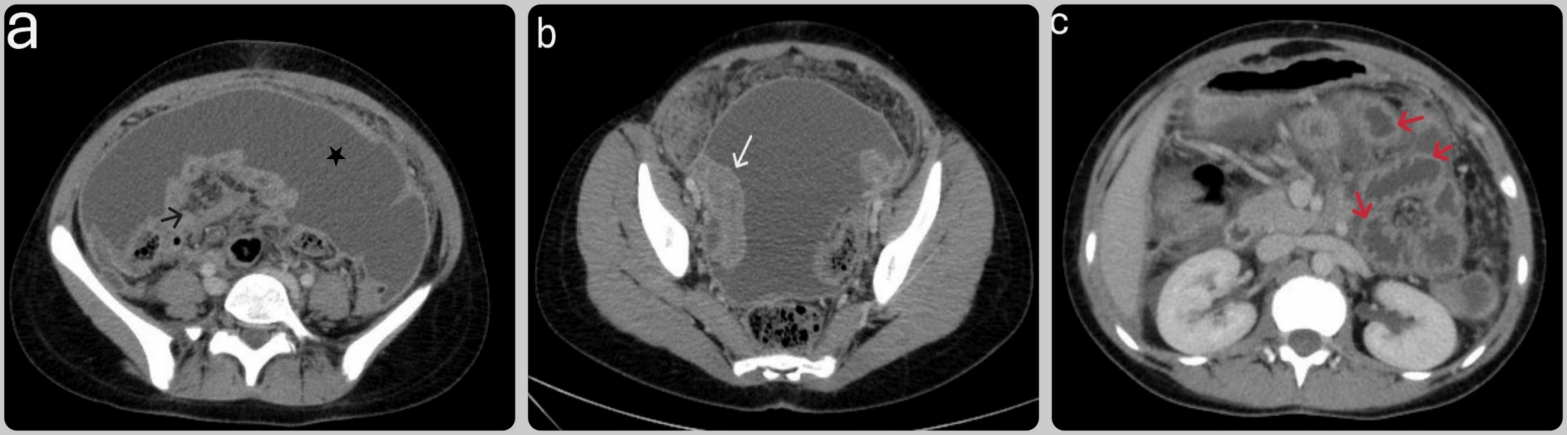
Abdomen CT scan (a) Axial CT scan of the abdomen showing diffuse nodular thickening of the mesentery (black arrow), with a thickened and enhanced peritoneum. Extensive ascites are visible as large fluid collections surrounding the abdominal organs (black star). (b) Axial CT scan of the pelvis demonstrating thickened and enhanced fallopian tubes (white arrow), raising the possibility of salpingitis. (c) CT scan image displaying fluid-filled, prominently congested jejunal loops (red arrows).

Following the biochemical evaluation, a computed tomography (CT) scan of the abdomen with intravenous (IV) contrast was performed. The imaging revealed extensive compartmentalized ascites with thickening of the enhancing peritoneum. Both ovaries were enlarged and contained masses, with multiple prominent mesenteric and portocaval lymph nodes noted (Figures 1a-1c).

Following imaging, tumor marker analysis was conducted to aid in differential diagnosis. The CA-125 level was markedly elevated at 449 U/mL (reference range: 0-35 U/mL), while lactate dehydrogenase (LDH) was slightly raised at 331 U/L (reference range of 140-280 U/L). Alpha-fetoprotein (AFP) was within the normal range at 0.75 IU/mL (reference range healthy <5.8), and beta-human chorionic gonadotropin (β-HCG) was <0.1 mIU/mL (reference range: 0-5 mIU/mL). These findings, in conjunction with the patient's clinical presentation, raised the suspicion of ovarian malignancy.

To further investigate, an ascitic fluid analysis was performed, showing a peritoneal albumin level of 2.8 g/dL, total protein at 5.34 g/dL, and a white blood cell count of 1,725 cells/μL with 58% neutrophils and 29% monocytes. Peritoneal TB culture was subsequently found to be negative.

These findings indicated exudative ascites, suggesting a malignant or infectious process rather than a transudative cause typically associated with portal hypertension. The elevated protein level and the high white blood cell count with a predominance of neutrophils further supported the likelihood of an underlying infection or inflammation, warranting additional investigation into potential infectious etiologies or inflammatory conditions.

**Figure 2:**
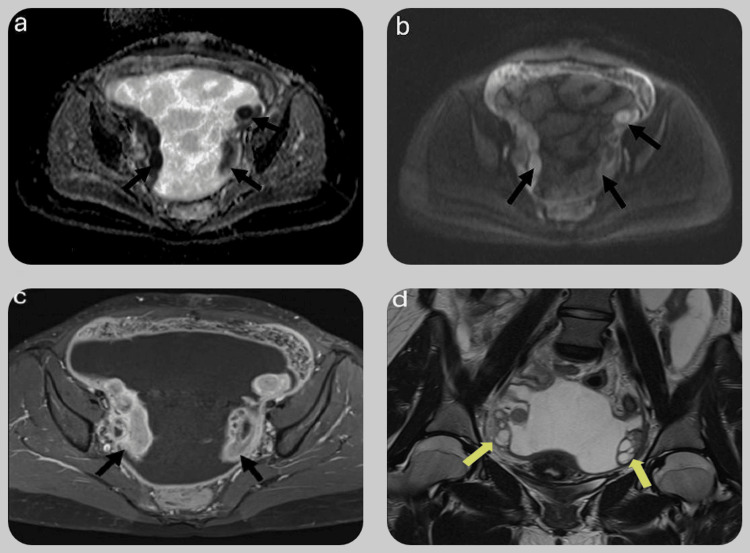
Pelvic MRI MRI pelvis images demonstrating three sequences: diffusion-weighted imaging (DWI) (a), apparent diffusion coefficient (ADC) (b), and post-contrast sequence (c). These images show intense mucosal enhancement of both fallopian tubes with restricted diffusion (black arrows), consistent with bilateral salpingitis. (d) Coronal MRI section showing normal bilateral ovaries (yellow arrows).

A gynecology consult was subsequently requested. To further evaluate the suspected pathology, a pelvic magnetic resonance imaging (MRI) with contrast was performed, which revealed a thickened, enhanced peritoneum. Bilateral salpingitis was observed, raising the possibility of TB peritonitis. However, the ovaries appeared normal on imaging (Figures 2a-2d).

A surgical consult was obtained, and the decision was made to proceed with an exploratory laparotomy. Intraoperative findings revealed marked thickening of the peritoneum with extensive intra-abdominal adhesions. Numerous nodules were noted on the omentum and peritoneum, with approximately 1.5 liters of fluid accumulation. Both the ovaries and uterus appeared normal during the procedure.

**Figure 3:**
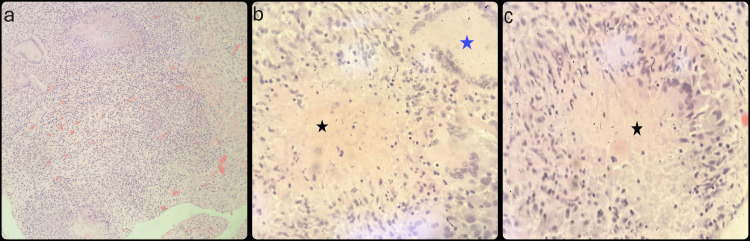
Histopathological findings (a) Low-power field showing mixed inflammatory cells within the tissue. (b) High-power field revealing a caseating granuloma (black star) alongside a non-caseating epithelioid granuloma with scattered Langerhans-type multinucleated giant cells (blue star). (c) High-power field demonstrating a caseating granuloma (black star).

**Figure 4:**
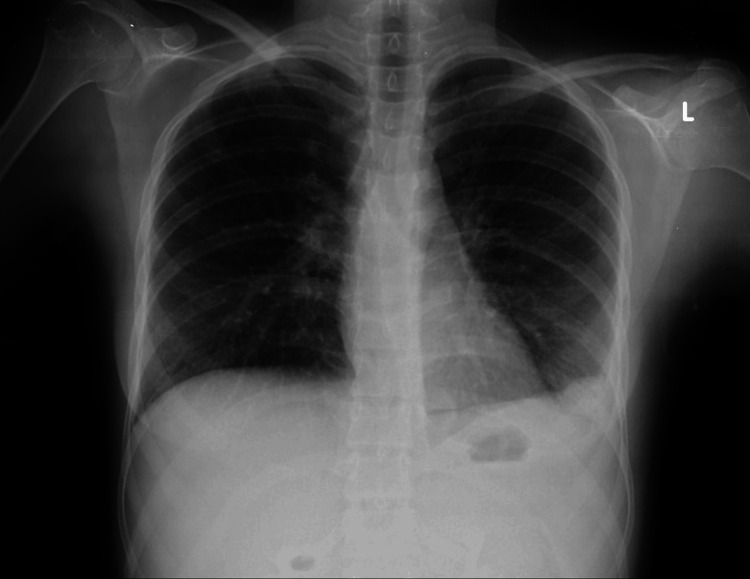
Chest x-ray

The postoperative course was uneventful. Histopathological examination of the omental biopsy and peritoneal fluid demonstrated signs of granulomatous inflammation, including caseating granulomas, which are indicative of TB (Figures 3a-3c). A postoperative chest x-ray (Figure 4) revealed no infiltrations. However, it showed a left-sided pleural effusion, which was deemed to be primarily reactive in nature.

The patient was diagnosed with extrapulmonary abdominal TB and initiated on anti-tubercular therapy. The treatment regimen consisted of four drugs for the first two months: rifampin (RIF) 600 mg (10 mg/kg), isoniazid (INH) 300 mg (5 mg/kg), pyrazinamide (PZA) (25 mg/kg), and ethambutol (EMB) (15 mg/kg). Thereafter, rifampin and isoniazid were continued for a total duration of nine months. Additionally, the patient received vitamin B6 (pyridoxine) 50 mg once daily to prevent isoniazid-induced neuropathy.

After eight months of follow-up, the patient demonstrated significant clinical improvement with a favorable response to the anti-tubercular therapy. She regained weight, reaching 67 kg, and her ascites resolved completely. She is scheduled to complete and discontinue treatment next month.

## Discussion

Abdominal TB can involve multiple anatomical locations, including the peritoneum, stomach, intestinal tract, hepatobiliary tree, pancreas, perianal region, and lymph nodes. The most commonly affected sites are the peritoneum, colon, and lymph nodes.

TB of the abdomen may arise from the reactivation of a dormant infection or through ingestion of *M. tuberculosis* via unpasteurized milk or undercooked meat. Moreover, in cases of active pulmonary TB or miliary TB, the abdomen may become involved via hematogenous dissemination, contiguous spread from adjacent organs (e.g., fallopian tubes), or lymphatic channels [6].

The clinical presentation of peritoneal TB includes ascites in 93% of cases, abdominal discomfort in 73%, and fever in 58% [7]. Symptoms typically persist for weeks to months before diagnosis [8-10]. Most patients with tuberculous peritonitis present with ascites at the time of diagnosis [11].

Radiographic imaging is crucial in suspected abdominal TB. CT, particularly with enterography protocols, is recommended for cross-sectional assessment of the intestines, other organs, and detection of ascites, peritoneal involvement, and lymphadenopathy [12-14]. CT imaging findings often include ascites, lymphadenopathy, thickened mesentery or omentum, and peritoneal thickening [15].

In this case, CT imaging showed features indicative of peritoneal TB. However, suspicion of ovarian cancer arose due to the presence of enlarged ovaries with masses. Subsequent MRI imaging confirmed normal ovaries. Distinguishing tuberculous peritonitis from peritoneal carcinomatosis can be challenging, as both conditions share imaging similarities. Key differences lie in ovarian capsular changes and parenchymal attenuation, which may aid differentiation [16].

In tuberculous peritonitis, the ascitic fluid typically has a protein concentration exceeding 3.0 g/dL and a cellular count ranging from 150 to 4,000 cells/μL, predominantly lymphocytes [17,18]. In this case, neutrophil-predominant ascitic fluid suggested a potential superimposed infection.

The diagnostic sensitivity of acid-fast bacilli (AFB) smear and mycobacterial culture in ascitic fluid is low (≤2% for AFB smear; ≤20% for culture). Broth culture results may take two to three weeks, while solid-phase cultures require even longer [19,20]. Although nucleic acid amplification testing (NAAT) for *M. tuberculosis* in ascitic fluid shows promise, its diagnostic efficacy remains uncertain. Some studies have reported a high detection rate using polymerase chain reaction (PCR) for TB DNA [21].

Measurement of peritoneal fluid interferon-gamma concentration may be a useful tool for diagnosis of tuberculous peritonitis [22]. However, this test does not have a role in routine evaluation of suspected tuberculous peritonitis and has not been approved by the US Food and Drug Administration for this purpose.

Laparoscopy offers high diagnostic accuracy, with a sensitivity of 93% and specificity of 98% for peritoneal TB [23]. Laparoscopic biopsy enables histological confirmation in approximately 82% of cases, while visual identification achieves 95% accuracy [24]. Characteristic laparoscopic findings include: Thickened peritoneum with or without yellowish-white lesions, adhesions and fibroadhesive patterns and caseating (necrotizing) granulomas, which are strongly indicative of TB though not definitive [25].

Peritoneal TB often mimics ovarian malignancy due to overlapping features such as ascites, peritoneal involvement, and elevated CA-125 levels. However, CA-125 elevation is nonspecific and may occur in both conditions [26,27]. Abdominal imaging, cytological analysis of ascitic fluid, and histological examination of biopsy samples are crucial to distinguish between benign and malignant causes.

The management of abdominal TB generally aligns with that of pulmonary TB, involving a standard six-month course of anti-TB therapy. This typically includes an initial intensive phase of four drugs (rifampin, isoniazid, pyrazinamide, and ethambutol) followed by a continuation phase [28-30]. However, in this case, a nine-month regimen was implemented, likely reflecting clinical judgment based on patient-specific factors, such as disease severity, response to treatment, or concerns about relapse risk.

This case illustrates the challenges in diagnosing abdominal TB, especially in differentiating it from ovarian malignancies. The overlap in symptoms, imaging findings, and laboratory markers highlight the importance of a multidisciplinary diagnostic approach, incorporating imaging, fluid analysis, and histological confirmation. This is particularly vital in younger patients, where timely diagnosis can prevent severe complications and ensure effective treatment.

## Conclusions

This case highlights the diagnostic challenges posed by abdominal TB, which can mimic ovarian malignancy in clinical presentation, imaging findings, and laboratory markers. The overlap underscores the importance of a thorough and multidisciplinary approach, including advanced imaging, ascitic fluid analysis, and laparoscopic biopsy, to establish an accurate diagnosis. Prompt initiation of appropriate anti-tubercular therapy led to a favorable outcome, with complete resolution of symptoms and ascites. This case emphasizes the need for heightened clinical suspicion of abdominal TB in regions where TB is endemic and illustrates the value of individualized treatment regimens tailored to patient response and disease characteristics.
